# NR4A1 enhances MKP7 expression to diminish JNK activation induced by ROS or ER-stress in pancreatic β cells for surviving

**DOI:** 10.1038/s41420-021-00521-0

**Published:** 2021-06-04

**Authors:** Ze-qing Pu, Tian-fu Yu, Dong Liu, Cheng-wen Jin, Esha Sadiq, Xiaofei Qiao, Xiaojie Li, Yuxuan Chen, Jinsong Zhang, Mingzhong Tian, Siying Li, Ru-xing Zhao, Xiang-dong Wang

**Affiliations:** 1 Department of Cell Biology, Shandong University School of Medicine, Jinan, China; 2 Shandong University School of Medicine, Jinan, China; 3 Laboratory of Human Genetics, Shandong Provincial Hospital (Dongying Branch), Affiliated to Shandong University, Dongying, China; 4 Department of Endocrinology, Qilu Hospital of Shandong University, Jinan, China

**Keywords:** Apoptosis, Type 2 diabetes

## Abstract

Under adverse conditions, such as sustained or chronic hyperglycemia or hyperlipidemia, ROS (reactive oxygen species) or/and ER-stress (endoplasmic reticulum stress) will be induced in pancreatic β cells. ROS or ER-stress damages β-cells even leads to apoptosis. Previously we found ROS or ER-stress resulted in JNK activation in β cells and overexpressing NR4A1 in MIN6 cells reduced JNK activation via modulating cbl-b expression and subsequent degrading the upstream JNK kinase (MKK4). To search other possible mechanisms, we found the mRNA level and protein level of MKP7 (a phosphatase for phospho-JNK) were dramatic reduced in pancreatic β cells in the islets from NR4A1 KO mice compared with that from wild type mice. To confirm what we found in animals, we applied pancreatic β cells (MIN6 cells) and found that the expression of MKP7 was increased in NR4A1-overexpression MIN6 cells. We further found that knocking down the expression of MKP7 increased the p-JNK level in pancreatic β cells upon treatment with TG or H_2_O_2_. After that, we figured out that NR4A1 did enhance the transactivation of the MKP7 promoter by physical association with two putative binding sites. In sum, NR4A1 attenuates JNK phosphorylation incurred by ER-stress or ROS partially via enhancing MKP7 expression, potentially decreases pancreatic β cell apoptosis induced by ROS or ER-stress. Our finding provides a clue for diabetes prevention.

## Introduction

In the past two decades, an increasing number of researchers were involved in research on the etiology of type 2 diabetes mellitus. It is popularly accepted that some adverse conditions, such as sustained or chronic hyperglycemia and/or hyperlipidemia, result in type 2 diabetes^1^. Hyperglycemia or hyperlipidemia usually causes cellular stresses, include ER-stress (endoplasmic reticulum stress) and/or oxidative stress associated with ROS (reactive oxygen species). It has been reported that pancreatic β cells will be damaged even lead to apoptosis when they suffer from cellular stresses^2^. But people should know that pancreatic β cells have a ‘protective system’ to resist these stresses. If the ‘protective system’ is not strong enough or loses its function, the pancreatic β cells will be in danger. To search on this ‘protective system’ is meaningful in finding clues for diabetes prevention.

NR4A1 is a multi-stress response factor, and it has the role of tackling some stresses in response^3–6^. Although it contains a ligand-binding domain, its natural ligand has not been found^7^. Therefore, it belongs to the orphan nuclear receptor^7,8^. The role of NR4A1 in apoptosis is still controversial. Sometimes it is anti-apoptotic and sometimes pro-apoptotic, depending on its localization. If NR4A1 remains in the nucleus as a transcriptional factor, it enhances the expression of some anti-apoptotic factors, such as WT1, survivin to anti-counter apoptosis;^9,10^ under some special conditions or challenged with some special drugs, NR4A1 is translocated into the cytoplasm, then NR4A1 associates with bcl2, which changes the confirmation and role of bcl2, finally it enhances apoptosis^11^. As an anti-stress factor, we hypothesized that NR4A1 could relieve some stresses and potentially save the cells from apoptosis induced by these stresses.

We found that either H_2_O_2_ (hydrogen peroxide, a kind of ROS) or TG (Thapsigargin, an ER-stress inducer) increased the accumulation of phosphorylated JNK in β cells^12^. And it was reported that the accumulated phosphorylated JNK leads to apoptosis^13–16^. Our previous data showed that in β cells, overexpression NR4A1 reduced the phosphorylated JNK level incurred by cellular stresses via enhancing cbl-b expression and subsequent MKK4 (mitogen-activated protein kinase kinase 4, a JNK kinase) protein degradation. To fully explore the possible mechanism of NR4A1 downregulating JNK phosphorylation, we also thought of the possibility that a specific phosphatase targeting phosphorylated-JNK might have a role in reducing p-JNK level as well. Mitogen-activated protein kinase phosphatases (MKPs) are a class of dual specific phosphatases, to remove the phosphate group from Thr and Tyr residues, to achieve negative regulation of JNK activation. We searched the available information online and found two possible p-JNK-targeting phosphatases (MKP2 and MKP7), which had putative NR4A1 binding sites in their promoter regulatory elements. We found the expression of MKP7 was largely reduced in pancreatic islets from NR4A1 KO mice. This study tried to determine if NR4A1 could enhance MKP7 expression and if MKP7 could reduce JNK activation in pancreatic β cells. We also studied the related mechanisms.

## Results

### NR4A1 knockout mice exhibits decreased MKP7 expression, abnormal insulin response, and increased JNK activation in the islets

**Fig. 1:**
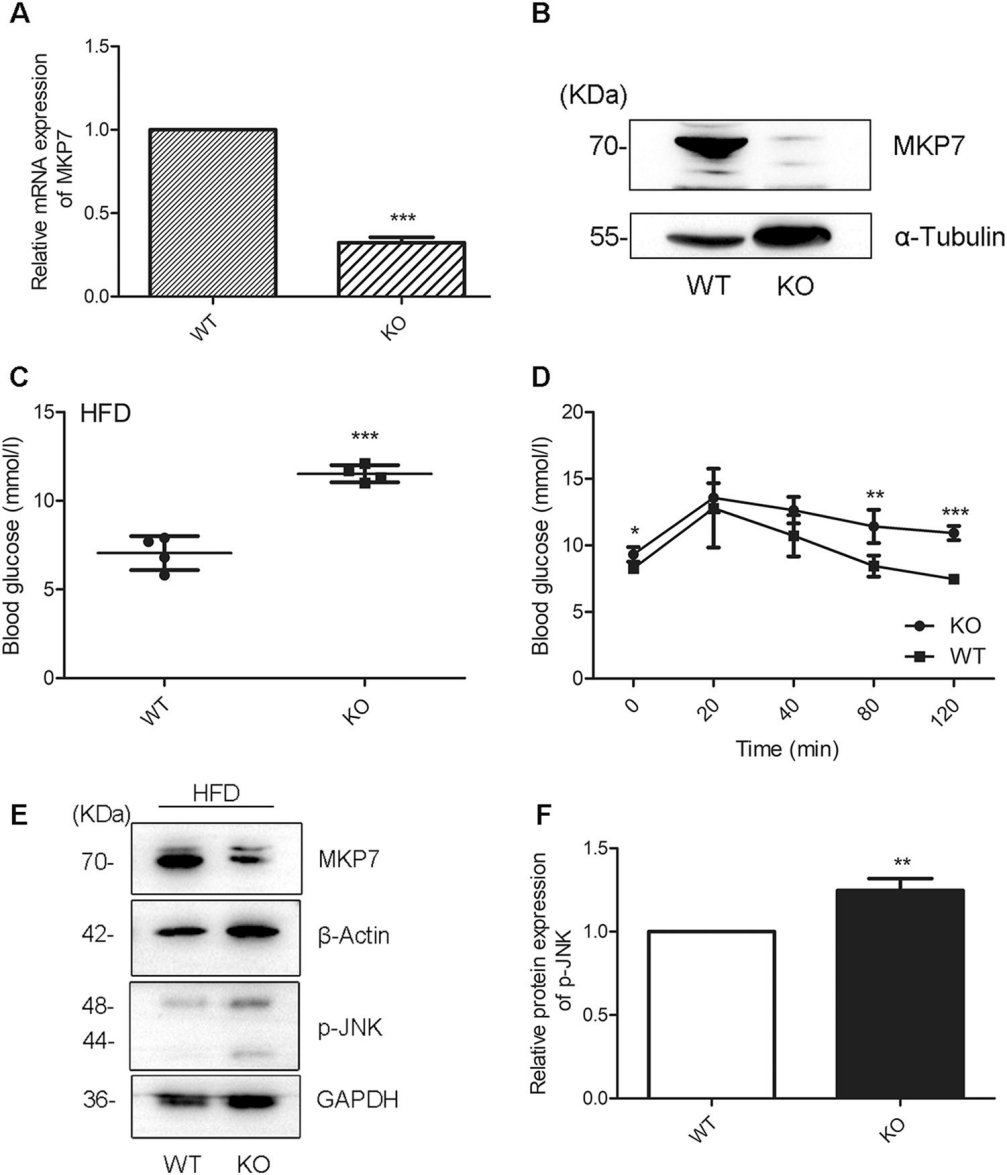
NR4A1 knockout mice exhibits decreased MKP7 expression, abnormal insulin response, and increased JNK activation. **A** The relative mRNA levels of MKP7 in the islets from WT and KO mice fed with normal diet were assayed with qPCR. **B** The relative protein level of MKP7 in the islets from WT or KO mice fed with a normal diet was obtained with Western Blotting. After 4 months of high-fat diet feeding, WT and KO mice were tested for the basal blood glucose level (**C**) and GTT (**D**). The relative p-JNK levels and the relative MKP7 levels in the islets from WT or KO mice fed with a high-fat diet for four months were analyzed (**E**–**F**). These data represented the means of three independent experiments, *P < 0.05, **P < 0.01, ***P < 0.001 vs. ns. Error bars were shown as SD values.

The real-time PCR data showed that the mRNA level of MKP7 was dramatically reduced in pancreatic islets from NR4A1 KO mice compared with that from WT mice fed with a normal diet (Fig. 1A). Western blotting results exhibited that the protein level of MKP7 was significantly reduced in the islets from NR4A1 KO mice compared with that from WT mice fed with normal diet (Fig. 1B). The pancreatic β cells account for 90% of the total cells in the islet in mice. Therefore, the q-PCR and western blotting data of pancreatic islets reflect almost the pancreatic β cells’ reality, at least 90%.

The NR4A1 KO mice and the control wild-type mice were fed with a normal diet for two months, then fed with a high-fat diet for three months. The results showed that KO mice had more elevated fasting blood glucose (Fig. 1C). Glucose tolerant test (GTT) showed that KO mice had defective glucose clearance ability, which meant the KO mice might not have enough insulin to reduce blood sugar (Fig. 1D).

We separated and purified the islets from WT or NR4A1 KO mice fed with a normal diet or with a high-fat diet. The protein samples were extracted from these islets. Western blotting results showed that there was no obvious difference regarding phospho-JNK level between the samples from WT and KO mice fed with normal diet (data not shown). While the phospho-JNK level was much higher in the sample from NR4A1 KO mice fed with a high-fat diet compared with that from WT mice (Fig. 1E–F). The MKP7 protein level was much lower in the islet from NR4A1 KO mice compared with that from WT mice fed with a high-fat diet (Fig. 1E) or a normal diet (Fig. 1B). These data indicated that a high-fat diet resulted in increased JNK activation in pancreatic islets from NR4A1 KO mice, while the MKP7 protein expression was reduced in the islets from NR4A1-KO mice compared with that from WT mice, which was independent of a high-fat diet.

### NR4A1 reduces JNK activation and apoptosis in pancreatic β cells incurred by ROS or ER-stress

**Fig. 2:**
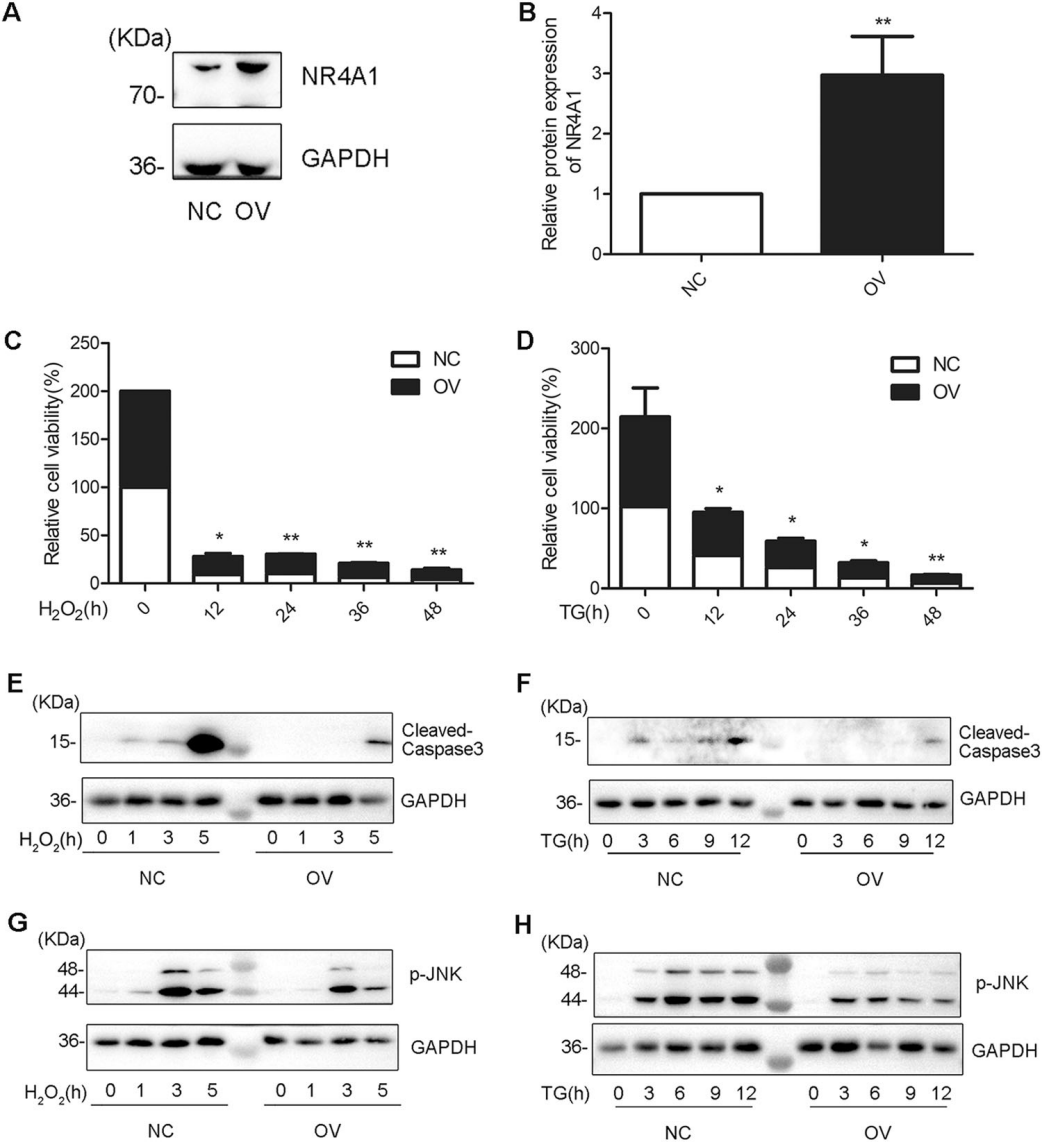
NR4A1 reduces apoptosis and JNK phosphorylation incurred by TG or H_2_O_2_ in MIN6 cells. **A**–**B** The relative NR4A1 protein levels in both OV (NR4A1 overexpression) and NC (control) cells were assayed with Western Blotting. **C**–**D** The relative cell viability in response to 100 μM H_2_O_2_ or 0.5 μM TG at various time points in both OV and NC cells was assayed with CCK8. **E**–**F** The protein levels of relative cleaved caspase-3 in response to 100 μM H_2_O_2_ or 0.5 μM TG at various time points in both OV and NC cells were obtained with western blotting. **G**–**H** The protein levels of p-JNK in response to 100 μM H_2_O_2_ or 0.5 μM TG at various time points in both OV and NC cells were shown in western Blotting. These data represented the means of three independent experiments, *P < 0.05, **P < 0.01 vs. ns. Error bars were shown as SD values.

We generated and cultured overexpressing NR4A1 cells (OV cells) or the control cells (NC cells) from MIN6 cells infected with lentivirus encoding NR4A1 cDNA or with a control lentivirus^10,11^. The protein expression levels of NR4A1 in OV cells and NC cells were confirmed with western blotting (Fig. 2A–B).

To confirm that NR4A1 has the role of protecting β cells from loss incurred by ER-stress or ROS, we analyzed the OV and NC cells’ viability. The OV and NC cells were seeded in 96 wells and treated with H_2_O_2_ or TG at a series of time points. Then, CCK8 analysis was applied to determine the viability of the two types of cells under ER or ROS stress. The data showed the viability of NC cells dropped much obviously than that of OV cells (Fig. 2C–D), which indicated that NR4A1 expression impacts cell viability in pancreatic β cells treated with H_2_O_2_ or TG. Namely, NR4A1 had a role in resisting pancreatic β-cell loss incurred by H_2_O_2_ or TG.

OV cells and NC cells were treated with TG at 0.5 μM or H_2_O_2_ at 100 μM at a series of time points. The cells were harvested for western blotting. During the treatment with H_2_O_2_, the active caspase-3 profile was different between OV and NC cells. It was clear that the active caspase-3 appeared and reached the peak much earlier in NC cells than in OV cells, which meant NR4A1 hindered MIN6 cells from apoptosis induced by H_2_O_2_ (Fig. 2E). Similarly, NR4A1 delayed MIN6 cells from apoptosis induced by TG (Fig. 2F).

We compared the JNK phosphorylation profiles and found overall the JNK phosphorylation level was lower in OV cells than that in NC cells, which indicated that overexpression NR4A1 turned down the JNK phosphorylation level in MIN6 cells (Fig. 2G–H).

### NR4A1 enhances MKP7 expression in pancreatic β cells

**Fig. 3:**
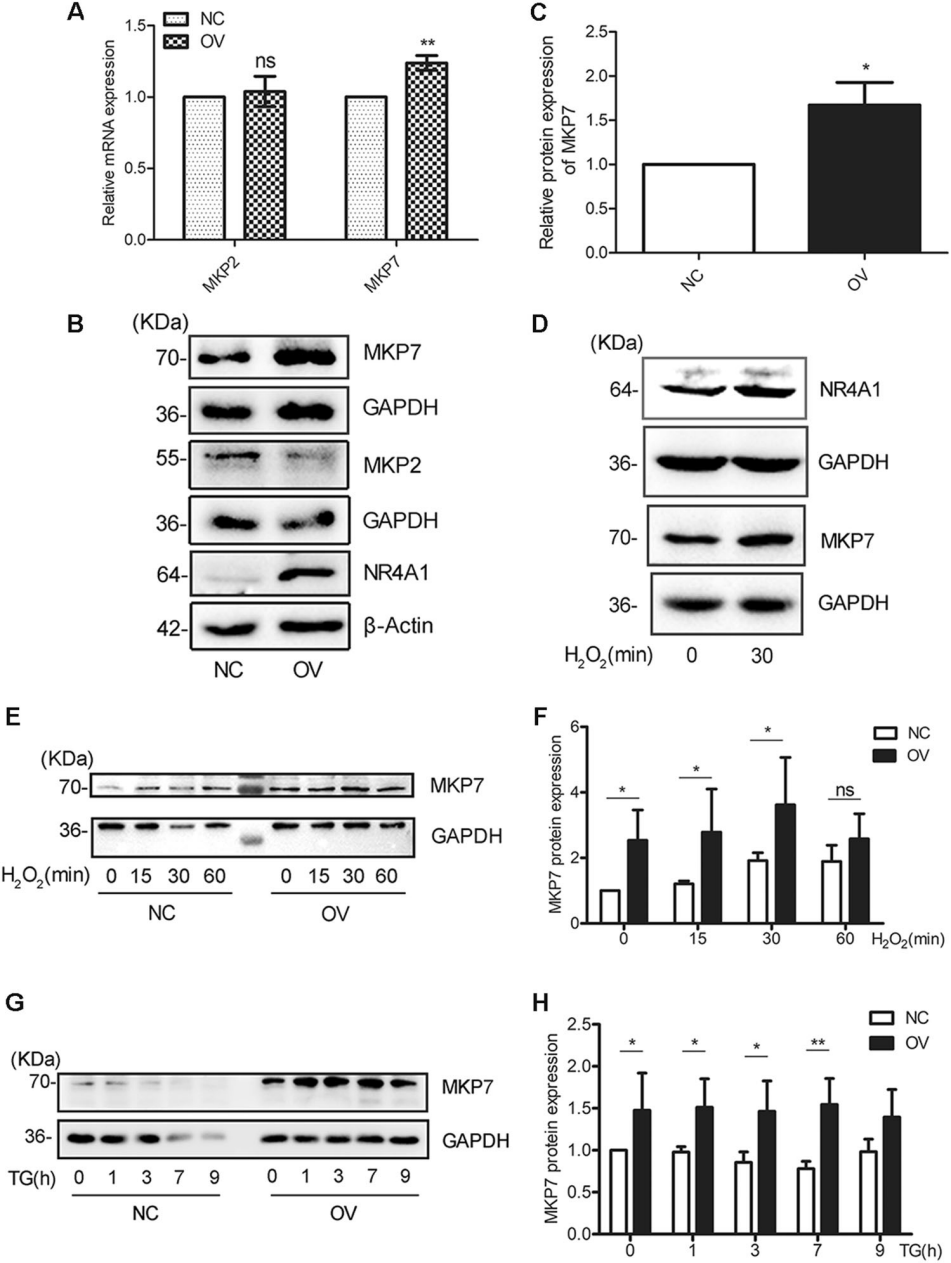
NR4A1 upregulates MKP7 expression. **A** The relative mRNA levels of MKP2 and MKP7 in NC and OV cells were determined with qPCR. **B** and **C** The relative MKP2 and MKP7 protein levels in both OV and NC cells were determined with Western Blotting. **D** The protein levels of MKP7 and NR4A1 in response to 100 μM H_2_O_2_ in NC cells. **E**–**H** The protein levels of MKP7 in response to 100 μM H_2_O_2_ and 0.5 μM TG at various time points in both OV and NC cells. The data represented the means of three independent experiments, *P < 0.05, **P < 0.01 vs. ns. Error bars were shown as SD values.

As we mentioned in the “Introduction” section, there were 2 phosphatases (MKP2, MKP7) targeting to p-JNK, and their promoter regulatory sequences had putative NR4A1 binding sites. We tested the mRNA levels of these three phosphatases in MIN6 cells overexpressing NR4A1 and found only the MKP7 mRNA level was significantly increased. While we did not find NR4A1 overexpression had an effect on the mRNA expression of MKP2 (Fig. 3A). Our data further exhibited that overexpression NR4A1 in MIN6 cells also increased the MKP7 protein level, rather than the MKP2 protein level (Fig. 3B–C).

MIN6 cells were treated with H_2_O_2_ for 30 min. The western blotting results showed that H_2_O_2_ increased MKP7 expression, accompanied by the increased NR4A1 expression (Fig. 3D).

During the drug treatment with H_2_O_2_ or TG for a series of time points, the MKP7 protein level was kept at a higher level in OV cells compared with that in NC cells (Fig. 3E–H), which meant that during the treatment, MKP7 was always available for its substrate.

### MKP7 expression negatively correlates with JNK activation incurred by ROS or ER-stress in pancreatic β cells

**Fig. 4:**
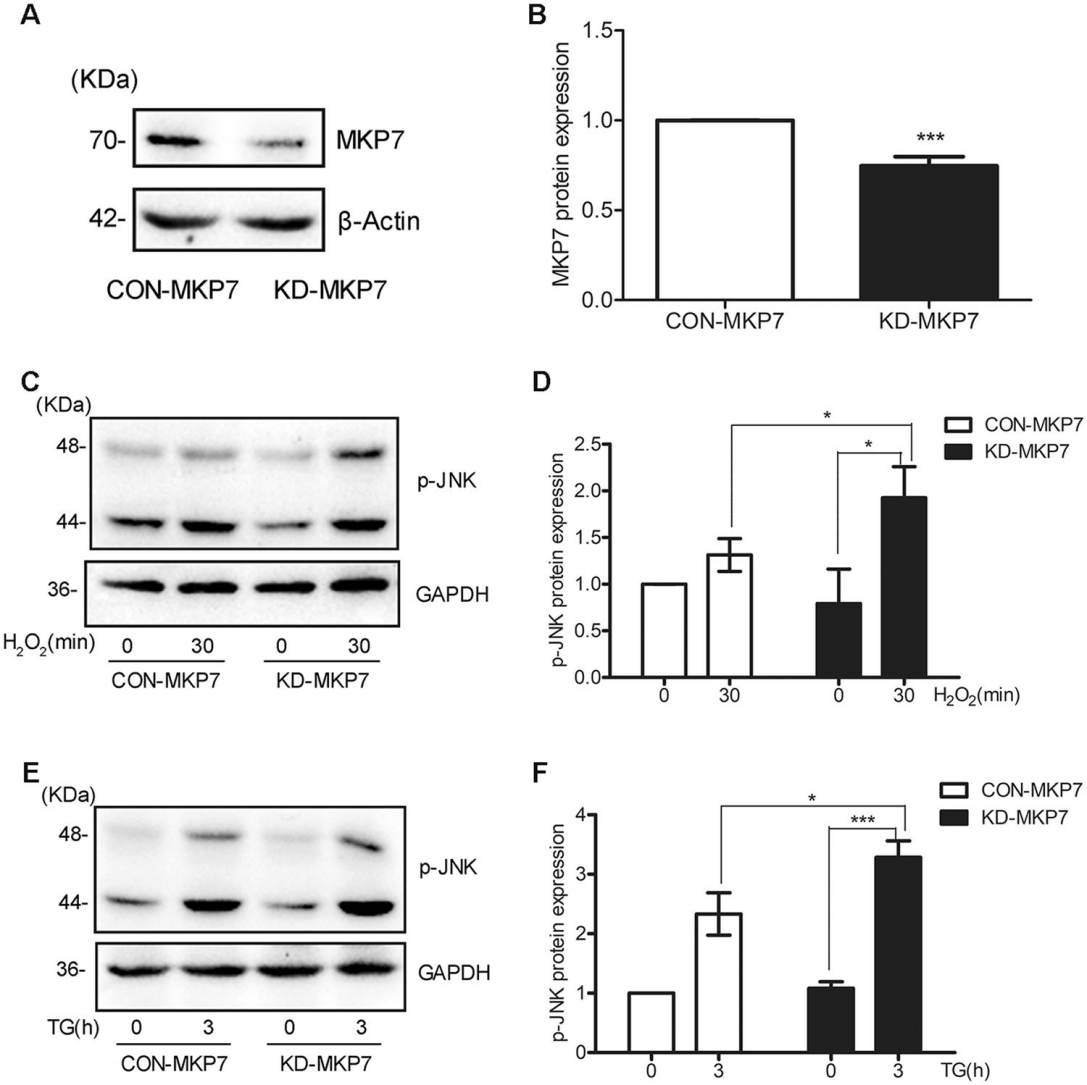
MKP7 expression negatively correlates p-JNK level under ER-stress or ROS conditions. **A**–**B** β-TC6 cells were infected with Lentivirus encoding shRNA targeting to MKP7 or with Lentivirus encoding a scrambled shRNA as control. The protein levels of MKP7 in KD-MKP7 (MKP7 knockdown) or CON-MKP7 cells were determined with Western Blotting. **C**–**F** The phosphorylated JNK (p-JNK) level in response to 100 μM H_2_O_2_ and 0.5 μM TG in KD-MKP7 cells and CON-MKP7 cells were analyzed with western Blotting. These data represented the means of three independent experiments, *P < 0.05, ***P < 0.001 vs. ns. Error bars were shown as SD values.

To figure out if MKP7 had an effect on JNK phosphorylation, we exploited lentivirus encoding shRNA targeting to MKP7 or Lentivirus encoding scrambled shRNA to infect β-TC6 cells. The western blotting result showed that the protein expression level of MKP7 was dramatic reduced in MKP7 knocked-down cells compared with the control cells (Fig. 4A–B).

The MKP7 KD cells and the control cells were treated with H_2_O_2_ or TG, and western blotting data showed that knocking down the expression of MKP7 resulted in increased JNK phosphorylation or activation (Fig. 4C–F).

### NR4A1 enhances the transactivation of MKP7 promoter via physical association

**Fig. 5:**
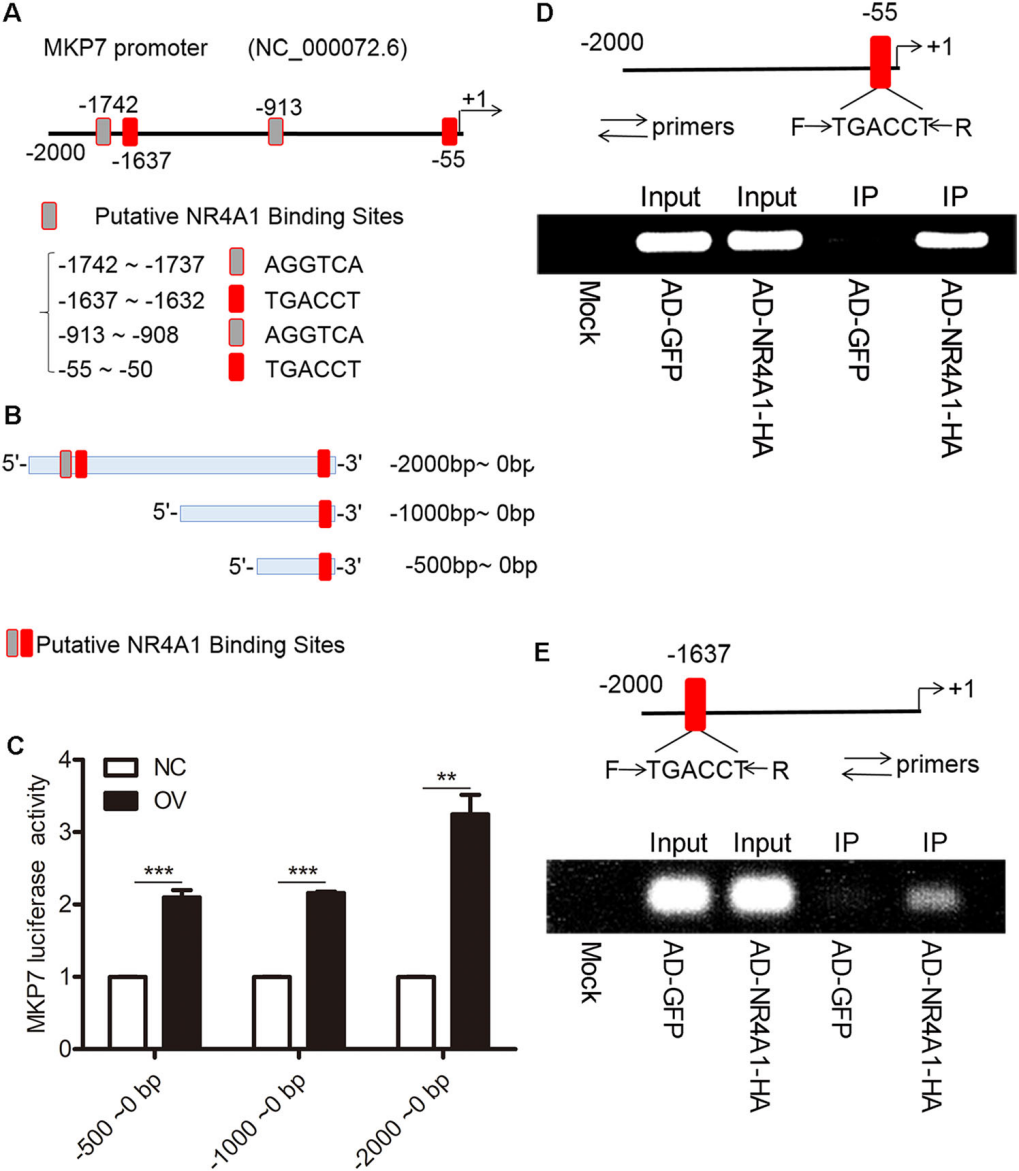
NR4A1 enhances the transactivation of the MKP7 promoter and physically associates with its promoters at two putative sites. **A** Sketch for NR4A1 putative binding site in MKP7 promoter. **B** A diagram of MKP7 promoters with different lengths was designed for luciferase reporter construction. **C** The relative luciferase activity of MKP7 promoters with different lengths exhibited in both OV and NC cells. **D** and **E** ChIP analysis were exploited to detect the physical association between NR4A1 and the promoter region of MKP7; the exogenous NR4A1-HA expression with adenoviral infection in MIN6 cells was associated with chromatin at some specific DNA sequences, after chromatin immunoprecipitation with anti-HA antibodies, the pulled-down DNA fragments were subjected to PCR analysis with specific pairs of primers. The two putative NR4A1 binding sites (−55 to −50, −1637 to −1632) in the MKP7 promoter regulatory sequence were confirmed with specific PCR amplification. These data represented the means of three independent experiments, **P < 0.01, ***P < 0.001 vs. ns. Error bars were shown as SD values.

The promoter sequence of MKP7 from 0 to −2000 and have 3 putative NR4A1 binding sites (Fig. 5A). We amplified 3 different lengths of MKP7 promoter and cloned these 3 promoters’ fragment into luciferase reporter vector to obtained 3 reporter plasmids, these fragments including: 0 to −500, 0 to −1000, and 0 to −2000 (Fig. 5B). These reporter plasmids were co-transfected with TK plasmids into OV or NC cells, respectively. The luciferase assay data are shown in Fig. 5C. From these data, we could see that NR4A1 enhanced the transactivation of the 3 reporters, but there was no much difference between reporter 1 and 2. Reporter 3 had more transactivation activity than reporter 1 or 2. Reporter 1 and 2 were close regarding the enhancement of MKP7 promoter transactivation by NR4A1.

We did the ChIP assay as described in the “Methods” section. The monoclonal anti-HA antibody was used to pull down adenoviral overexpressed NR4A1-HA and its associated DNA fragments. Figure 5D–E showed that two pairs of primers designed to amplify the fragments covered the two putative NR4A1 binding sites in the promoter sequence. Figure 5D showed that the first putative NR4A1 binding site was successfully amplified with PCR from the ChIP pull-down product; Fig. 5E showed the third putative NR4A1 binding site was successfully amplified from the ChIP pull-down.

### Summary of our data

**Fig. 6:**
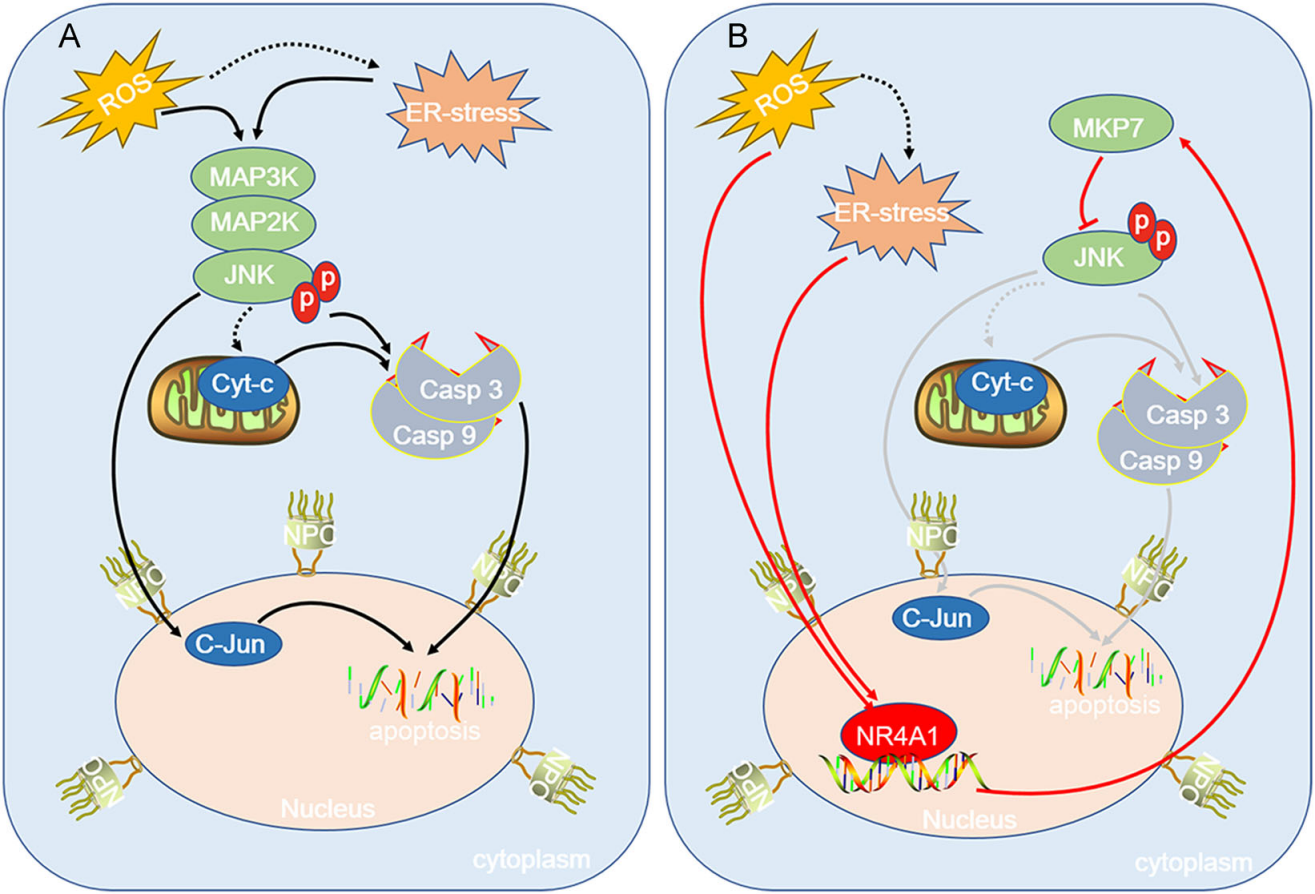
A model for NR4A1 regulating MKP7 expression and pancreatic β cell protection. **A** ROS may result in ER-stress. ROS or/and ER-stress incurs JNK activation. The activated JNK leads to pancreatic β cell apoptosis via mitochondria-dependent or independent pathways. **B** Acute ROS or ER-stress results in the expression of NR4A1, which in turn enhances the expression of MKP7, while MKP7 is able to abolish partial JNK activation by exerting its phosphatase activity, therefore, largely reduces pancreatic β cell apoptosis. The fate of pancreatic β cells depends on the balance between the availability of NR4A1 or MKP7 and the severity of ROS or ER-stress.

Figure 6 is the work model of our data. Sustained hyperglycemia or hyperlipidemia results in ROS and ER-stress in β cells. ROS is able to induce ER-stress. ROS or ER-stress leads to JNK activation, a high level of activated JNK results in apoptosis of β cell via mitochondria-dependent or independent pathways. Meanwhile, acute ROS or ER-stress is able to induce the expression of NR4A1; as a transcription factor, NR4A1 enhances the expression of MKP7, which is a phosphatase for phospho-JNK. MKP7 reduces activated JNK level, which turns down the possibility of β cell apoptosis incurred by JNK activation. The fate of pancreatic β cells lies in the balance between the availability of the MKP7 molecule and the severity of ROS or/and ER-stress.

## Discussion

People took a lot of effort to explore the mechanisms of type 2 diabetes formation for the cases of type 2 diabetes in the population increasing rapidly, and type 2 diabetes mellitus has been becoming a severe hazard to society’s health. So far, the mechanism of type 2 diabetes mellitus formation has not been fully deciphered. But some agreements have been reached that hyperglycemia or hyperlipidemia induces ER-stress and ROS in pancreatic β cells;^17–24^ if there is no protective system or molecules in β cells, then the β cells will be in danger or even be challenged to apoptosis or cell loss. We believe that there is a ‘protective system’ in pancreatic β cells to extinguish the ‘fire’ from ROS and ER-stress. In fact, our pancreatic β cells always face the challenge of ROS and ER-stress after the meal, but our pancreatic β cells always survive over these stresses owing to the healthy ‘protective system’. As we described before, NR4A1 and its downstream molecules might form such a ‘protective system’ to protect β cells from apoptosis or loss^12^.

Our previous studies showed that ROS (H_2_O_2_) or ER-stress inducer (TG) induced MIN6 cells apoptosis at some dose or time duration^10,11^. The mechanisms of ROS or ER-stress inducing apoptosis of β cells are complex and well described in many literatures. We reported that H_2_O_2_ or ER-stress induced JNK activation in MIN6 cells^12^. It was reported earlier that H_2_O_2_ triggered ER-stress^25–29^. More reports indicated that ROS or ER-stress resulted in JNK phosphorylation in different cells^30–32^ and subsequent apoptosis^26,33–39^. JNK phosphorylation inhibitor could partially reverse the ER-stress or ROS-induced apoptosis^40^, which indicated JNK activation closely related to apoptosis induced by ROS or ER-stress. In a word, ROS and ER-stress are two different stresses, but they can induce JNK activation and further apoptosis in pancreatic β cells as we confirmed in this report.

JNK belongs to the mitogen-activated protein kinases (MAPK) superfamily and has three isoforms (JNK1, JNK2, and JNK3). Many signal transduction pathways trigger JNK activation and the downstream signaling will negatively feedback to reduce the activated JNK back to normal, thus the homeostasis of the cell will be resumed. Appropriate JNK activation is required in the cells to keep some activities. But sustained high level of JNK activation will trigger apoptosis cascade. ER-stress or ROS enhances a high level of JNK activation, which may exceed the threshold for cell death. The molecules that reduce JNK activation will protect the cells from apoptosis induced by various stresses.

As it is reported, NR4A1 (also named Nur77) is a multi-stress response molecule to reduce the effect of stress^3–6^. We previously reported that ER-stress or H_2_O_2_ was able to induce the expression of NR4A1^10–12^. We have confirmed that exogenous NR4A1 over-expressed in MIN6 cells (OV cells) is located in the nucleus rather than cytosol and NR4A1 is able to protect beta cells from apoptosis by ER-stress or H_2_O_2_ via enhancing the expression of its downstream molecules or genes^9,10^. As a transcription factor, NR4A1 is able to enhance cbl-b expression, while cbl-b is an E3 ligase targeting MKK4 for proteasomal degradation. It is known that MKK4 is an upstream kinase for JNK. Therefore, NR4A1 is able to reduce JNK phosphorylation by ER-stress or ROS via an indirect way^12^.

To fully explore other possible mechanisms of NR4A1 downregulating JNK phosphorylation, we thought that it was also possible for NR4A1 to enhance the protein level of some phosphatase specific for phosphorylated-JNK. MKPs can be divided into three groups: the first group is the inducible nuclear phosphatase DUSP1/MKP1, DUSP2/PAC1, DUSP4/MKP2, and DUSP5/hVH3. Except for DUSP5, these MKPs showed a wide range of specificities for the inactivation of p38, JNK, and ERK. The second group contains three ERK-specific cytoplasmic MKPs, DUSP6/MKP3, DUSP7/MKPX, and DUSP9/MKP4. The third group consists of DUSP8/hVH5, DUSP10/MKP5, and DUSP16/MKP7, all of them work preferentially on phospho-p38 and phospho-JNK activated by cellular stresses^41^. We analyzed the possible phosphatases targeting to p-JNK. We obtained the promoter sequences of these genes from PubMed (gene bank) and found MKP2 and MKP7 had NR4A1 putative binding sites in their promoter sequences. MKP7 had 4 NR4A1 putative binding sites and these sites were closer to the critical promoter sequence. Therefore, we tested the mRNA and protein expression of MKP7 in the islets from NR4A1 KO mice and WT mice and found the protein level of MKP7 was largely reduced in the islets from NR4A1 KO mice compared with that from WT mice. As we mentioned in the “Results” section, the majority of cells in mice islets are pancreatic β cells (more than 90%). Therefore, the data from islets reflect the reality of pancreatic β cells. Later on, this conclusion was further confirmed by our in vitro study with the pancreatic β cell line.

MKP7, also known as dual-specificity phosphatase 16 (DUSP16), participates in cell activities. The roles of DUSP 16 in tumor development are controversial depending on different cell types. People found that DUSP16 as JNK phosphatase can suppress cell growth and transform BCR-ABL Rat-1 cells in vitro and in vivo^42^. While people also found that DUSP16 had a role in pro-tumor development^43^. MKP7 might have a positive role in metabolism. It was reported that DUSP16 could directly interact with TAK1 (Transforming growth factor b (TGF-b)-activated kinase) and negatively regulate JNK signaling to alleviate metabolic stress-induced hepatic steatosis^44^. Acute oxidative stress restored normal insulin sensitivity and glucose uptake in insulin-resistant muscle cells, and this effect was dependent on MKP7. Chronic oxidative stress resulted in increased levels of p-JNK in the cytoplasm, whereas acute oxidative stress led to redistribution of MKP7 from the nucleus into the cytoplasm, reduction in cytoplasmic p-JNK, and a concurrent accumulation of p-JNK in the nucleus^45^. It was reported that adenoviral overexpression of MKP-1 and MKP-7 prevented the phosphorylation of JNK and decreased MIN6 cell death^46^. We tried to bridge the connection among NR4A1, MKP7, and JNK activation in pancreatic β cells challenged with ER-stress or ROS.

To confirm what we found in the animal study was real, we applied MIN6 cells overexpressing NR4A1 for more tests. Our data showed that overexpression NR4A1 in MIN6 cells resulted in the increased expression of MKP7 at both mRNA and protein levels. H_2_O_2_ induced the expression of NR4A1 accompanied by increased MKP7 expression. To study if MKP7 has a role in downregulating cellular stress-induced JNK phosphorylation, we knocked down MKP7 expression in β-TC6 cells and found MKP7 expression was negatively correlated with p-JNK level. This result indicates that MKP7 has a role in modulating JNK phosphorylation in β cells challenged with cellular stress inducers. At this point, we conclude that NR4A1 is closely associated with MKP7 expression, and MKP7 expression impacts JNK activation incurred by ER-stress and ROS.

To study how NR4A1 upregulates MKP7 expression, we applied luciferase assay and ChIP assay. We amplified 3 different lengths of MKP7 promoter sequences (0 to −2000, 0 to −1000, and 0 to −500) and cloned them into luciferase reporter vector, and these reporter sequences covered 4, 2, 1 putative NR4A1 binding sites. It seemed that there was not much difference between the first reporter (0 to −500) and the second reporter (0 to −1000) regarding the MKP7 promoter transactivation by NR4A1, which meant the putative binding site (−913 to −908, AGGTCA) had no much impact on promoter transactivation. Therefore, we focused on the putative site as TGACCT (−55 to −50 and −1637 to −1632). Our ChIP assay indicated that NR4A1 was physically associated with these two putative binding sequences. So we conclude that NR4A1 enhances MKP7 promoter transactivation via direct associating with two putative targeting sequences in the promoter.

In summary, our data demonstrate that NR4A1 attenuates ER-stress and ROS-induced JNK activation via enhancing MKP7 expression in pancreatic β cells, therefore, the risk of pancreatic β cell loss will be largely reduced. The results of this study enrich the knowledge of NR4A1 downregulating JNK over-activation and provide clues for β cell protection or even diabetes prevention. Seeking ways or drugs to enhance NR4A1 expression in our body would be helpful to reduce the possibility of diabetes development.

## Methods

### Cell culture and reagents

The mouse pancreatic β cell lines (MIN6 and β-TC6) were from ATCC. NR4A1 overexpression cell lines (designated as OV) and control cell lines (designated as NC) were generated from MIN6 cells. MKP7 knockdown cells (defined as KD-MKP7 cells) or the control cell clone (defined as CON-MKP7 cells) were generated from β-TC6 cells. MIN6 and β-TC6 cells were cultured with high glucose DMEM (CM15019/10013, M&C GENE TECHNOLOGY), supplemented with 10% Fetal Bovine Serum (A3160801, Gibco), 70 μM β-mercaptoethanol (M3148, Sigma-Aldrich), 100X penicillin-streptomycin (CC004, M&C GENE TECHNOLOGY) in a humidified environment (95% air and 5% CO_2_) at 37 °C. OV cells and NC cells were cultured in the same medium as MIN6 cells plus puromycin at 2 μg/ml. MKP7 knockdown cells and the control cell clone were cultured in the same medium as β-TC6 cells plus neomycin (G418) at 1 mg/ml.

### Determination of cell viability

The cells were seeded in a 96-well plate (100 μl/well) and cultured for 24 h. Blank wells and control wells were set at the beginning. H_2_O_2_ or Thapsigargin (TG) were added into the cells at different time points at fixed final concentrations. After that, the cells were incubated at 37 °C for a different time duration, then 10 μl CCK-8 Solution was added to each well. After 4 h additional incubation, the absorbance at 450 nm was detected with a microplate reader. The relative viability of each condition was calculated by using the following formula: Relative Viability = [(A − C)/(B − C)] × 100% [A: Absorbance value of the experimental group (this absorbance value comes from the medium, the cells, the test drug, and CCK-8 Solution); B: Absorbance value of the control group (the absorbance value comes from the medium, the cells, and CCK-8 Solution); C: Absorbance value of blank group (the absorbance value comes from the medium and CCK-8 Solution)].

TG was purchased from Sigma (SML1845), the stock solution was dissolved in DMSO. H_2_O_2_ was purchased from Sinopharm Chemical Reagent (10011218). When applied, it was diluted in pure water.

### Mouse Islets separation and purification

NR4A1 knockout (KO) mice and wild-type (WT) mice were purchased from Cyagen Biosciences. NR4A1 knockout (KO) mice generated from C57BL/6N mice. The newborn mice’s genotypes were confirmed with PCR by using the specific primers provided by the manufacturer. Being raised for two months old, the WT or KO mice with similar body weights and sound health were selected for random grouping, there were 12 to 15 mice in one group. Then the grouped mice were fed with a normal diet or high-fat diet (Fat content 60%) for 4 months. During this period, if the individuals became sick, they would be removed from the group. Then the mice pancreatic islets were isolated from the pancreas with Collagenase P (Roche) digestion via vein injection, the flow-out pancreas tissues with some islets were further digested at 37 °C for 20 min. Then the digestion was terminated with Hanks Solution; after that, the islets were subjected to sinking to the bottom of a culture dish by gravity force. Finally, the islets were collected under the stereoscope. In each experiment, three WT or KO mice were selected randomly for islet purification, the mixed islets from the three WT or KO mice were applied for analysis of mRNA or protein expression. All the experiments were repeated three times.

Animal experiments were carried out in accordance with the Principles of Laboratory Animal Care established by the National Institutes of Health (USA). All experiment procedures were approved by the Animal Care and Use Committee of Shandong University.

### Mice blood glucose determination and glucose tolerant test (GTT)

The mice were labeled and starved overnight (approximately 16 h). Then the body weight (B.W) of each mouse was measured. The fasting blood glucose was measured with a blood glucose meter. GTT was carried out by injecting the mouse intraperitoneally with glucose at 1 mg/g (B.W). After glucose injection, the blood sugar was measured at 20 min, 40 min, 80 min, and 120 min post glucose injection.

### Generating stable cell lines with lentiviral infection

Obio Technology reconstructed lentivirus encoding shRNA targeting MKP7 and control lentivirus encoding scrambled shRNA. The following siRNA sequence 5′-GCAACAGGACAAAGTATTA-3′ was utilized for the MKP7 targeting sequence. β-TC6 cells were infected with MKP7 shRNA lentivirus or control scramble shRNA lentivirus; after that, stable cells were selected under neomycin (G418) drug pressure. Western blotting and qPCR analysis were used to confirm that MKP7 protein expression was largely reduced.

### Luciferase reporter plasmids construction

Mice genomic DNA was extracted with ReverTra Ace qPCR RT Kit (Toyobo). We also amplified the MKP7 promoter sequences (2000bp, 1000 bp, 500 bp) from mice genomic DNA by using the designed primers with PCR, and then cloned the PCR products into pGL3-Basic luciferase reporter vector. All constructed plasmids were sequenced by Sangon Biotech Co., Ltd. (Shanghai, China) to ensure these sequences were completely aligned with the original DNA sequences from NCBI. Three pairs of primer sequences with Kpnl (GGTACC) and SacI (GAGCTC) restriction sites were designed as follows:

Primer F (−2000bp): 5′-CCC GGTACC GAGTGCCAGAATTGTCTTGCTA-3′

Primer F (−1000bp):

5′-CCC GGTACC ACCGATGTCGAGAATACTTACTTAATG-3′

Primer F (−500bp): 5′-CCC GGTACC CCTGTAACTTCCCTCTCAAGAGAA-3′

Primer R (0 bp): 5′-CCC GAGCTC GAGAAAGAGTCGCTGGTCAGGAAA-3′

### Dual-luciferase reporter assays

Luciferase reporter plasmids with MKP7 promoter were co-transfected into MIN6 cells (OV or NC cells) with TK (thymidine kinase promoter-Renilla luciferase reporter) plasmids, or pGL3-Basic vectors were co-transfected into MIN6 cells with TK plasmid as a control. The luciferase activity was measured by a dual-luciferase reporter assay kit (Beyotime) after 48 h post-transfection. Renilla luciferase was used as the internal control; the RLU value obtained by measuring the firefly luciferase activity was divided by the RLU value obtained by measuring Renilla luciferase activity. The relative luciferase activity enhancement in OV cells vs. NC cells reflected the transactivation of the MKP7 promoter impacted by NR4A1.

### Chromatin immunoprecipitation (ChIP) analysis

The cultured MIN6 cells were infected with adenovirus encoding NR4A1-HA or control adenovirus. After 48 h post-infection, the infected cells were applied for ChIP assay. ChIP analysis was accomplished with a ChIP Assay kit (Beyotime). The experiments were carried out according to the manufacturer’s instructions. During this process, the genomic DNA was broken up to 200–1000 bp fragments by sonication. The antibody applied for HA-tagged NR4A1 was an anti-HA monoclonal antibody (12CA5), purchased from Roche. The pull-down DNA products were used for PCR analysis with specific primers. Agarose gel electrophoresis was used to detect if NRA1 could associate with particular promoter regulatory elements in genomic DNA.

The following two pairs of primers were, respectively, designed to amplify the specific targeting sequences of −86 bp to −17 bp and –1663 bp to −1534 bp in the MKP7 regulatory region.

5′-GGATTGGTTTCAAGTGACGCCATCTC-3′ (F),

and 5′-GAGAAAGAGTCGCTGGTCAGGAAACTTC-3′ (R);

5′-GGGCTCCCTTTTTCACTCTGAAAGATGACC-3′ (F),

and 5′-GTCCTCTCCCACCTTTAAACCCTGCAAC-3′ (R)

### Quantitative real-time PCR assay

Total RNA was extracted and purified from cultured cells using RNAiso Plus (Takara) and from mouse islets using an RNeasy Mini kit (Qiagen). Then reverse transcription of RNA to cDNA with ReverTra Ace® qPCR RT kit. qPCR was performed by using UltraSYBR Mixture and CFX96 Real-Time System of BIO-RAD. According to the Cq value of the target gene, the relative expression multiple of the target gene was calculated with a comparative quantitative method for 2^−ΔΔCq^, the numerical value was further corrected by using the housekeeping gene 18s rRNA as the internal control. All experiments were repeated three times, and there are three replicates at a time.

The primers used for PCR are listed in Table 1.

**Table 1:** For real-time quantitative PCR.

Genes	sequence
18S rRNA F	CGCGGTTCTATTTTGTTGGT
18S rRNA R	AGTCGGCATCGTTTATGGTC
NR4A1 F	ATGCCTCCCCTACCAATCTTC
NR4A1 R	CACCAGTTCCTGGAACTTGGA
MKP2 F1	TCCCCGTCGAAGACAACCA
MKP2 R1	CTTTACTGCGTCGATGTACTCG
MKP7 F1	AAGTGCTGCTAATTGATAGCCG
MKP7 R1	TGTCAACCTTATGCTTTGCAGAA

### Western blot analysis

The harvested cells were lysed with RIPA Lysis Buffer (Strong) plus protease inhibitor cocktail. The supernatant was collected after high-speed centrifugation and was stored for western blotting. The western blotting was carried out according to the methods routinely used. The islets were lysed with RIPA Lysis Buffer (Strong) plus protease inhibitor cocktail. The samples were obtained according to the manufacture’s instruction and applied for western blotting.

The antibodies used in the experiment were as follows: cleaved caspase-3 Antibody (9661) and rabbit anti-pJNK antibody (4668) were purchased from CST; mouse anti-GAPDH (10494-1-AP), rabbit anti-MKP2 antibody (10739-1-AP), and rabbit anti-MKP7 antibody (14237-1-AP) were derived from Proteintech Group (Wuhan, China); rabbit anti-NR4A1 antibody (ab13851 and DF7850) was purchased from Abcam and Affinity; mouse anti-β-Actin (BS6007M) and rabbit anti-tubulin α (BS1699) were purchased from Bioworld Technology.

### Statistical analysis

Data were expressed as the mean ± SD (standard deviation). *t*-test or analysis of variance (ANOVA) in GraphPad Prism 5.0. A value of *P* < 0.05 (*) was considered a statistically significant difference.
